# miRNA expression analysis in the human heart: Undifferentiated progenitors vs. bioptic tissues—Implications for proliferation and ageing

**DOI:** 10.1111/jcmm.16824

**Published:** 2021-08-13

**Authors:** Gioacchin Iannolo, Maria Rita Sciuto, Nicola Cuscino, Claudia Carcione, Claudia Coronnello, Cinzia Maria Chinnici, Giuseppe Maria Raffa, Michele Pilato, Pier Giulio Conaldi

**Affiliations:** ^1^ Department of Research Istituto Mediterraneo per i Trapianti e Terapie ad alta specializzazione (ISMETT‐IRCCS) Palermo Italy; ^2^ Department of Oncology and Molecular Medicine Istituto Superiore di Sanità Rome Italy; ^3^ Fondazione Ri.MED Palermo Italy; ^4^ Cardiac Surgery and Heart Transplantation Unit Department for the Treatment and Study of Cardiothoracic Diseases and Cardiothoracic Transplantation Istituto Mediterraneo per i Trapianti e Terapie ad alta specializzazione (ISMETT‐IRCCS) Palermo Italy

**Keywords:** cardiac repair, cardiovascular, Heart, miRNAs, myocardial infarction, NGS

## Abstract

In developed countries, cardiovascular diseases are currently the first cause of death. Cardiospheres (CSs) and cardiosphere‐derived cells (CDCs) have been found to have the ability to regenerate the myocardium after myocardial infarction (MI). In recent years, much effort has been made to gain insight into the human heart repair mechanisms, in which miRNAs have been shown to play an important role. In this regard, to elucidate the involvement of miRNAs, we evaluated the miRNA expression profile across human heart biopsy, CSs and CDCs using microarray and next‐generation sequencing (NGS) technologies. We identified several miRNAs more represented in the progenitors, where some of them can be responsible for the proliferation or the maintenance of an undifferentiated state, while others have been found to be downregulated in the undifferentiated progenitors compared with the biopsies. Moreover, we also found a correlation between downregulated miRNAs in CSs/CDCs and patient age (eg miR‐490) and an inverse correlation among miRNAs upregulated in CSs/CDCs (eg miR‐31).

## 1 INTRODUCTION

The leading cause of morbidity and mortality in developed countries is cardiovascular disease (CVD).^1^ Heart development in mammals is mainly dependent on cardiomyocyte (CM) proliferation,^2^ and after birth the replicative activity is reduced, with a slight renewal during adult life to repair cardiac damage in pathological conditions. Cardiomyocyte proliferation is essential for cardiac repair, but causes cardiac dysfunction when damage to cardiomyocytes occurs.

The mechanisms that regulate the self‐renewal ability in cardiomyocytes operate under high regulation. This regulation has been found to be coordinated by, among others, microRNAs (miRNAs), which have been shown in recent years to play a key role in both the development and repair processes.^3^ Preserving CM activity is the principal challenge in acute myocardial infarction (AMI) given that necrosis, a consequence of the ischaemic process, is difficult if not impossible to cure or prevent from causing permanent damage to CMs. The current therapy involves the administration of thrombolytic drugs, or heart transplantation, which is restricted by organ availability and donor age. In this respect, miRNAs may represent a valid therapeutic option for cardiac disease (revised by Hudson and Porrello^4^).

miRNAs are small noncoding RNAs of about 22 nucleotides (mature miRNAs) that regulate the post‐transcriptional expression of several genes. After the transcription, the primary miRNA transcripts are 5’ capped and 3’ polyadenylated in the nuclei and processed by Drosha‐DGCR8 complex to pre‐miRNAs hairpin loop structure (canonical pathway) or by the spliceosome to mirtrons through direct splicing of mRNA introns and refolded into a pre‐miRNA (alternative pathway). The pre‐miRNAs are exported in the cytoplasmic region by exportin 5 and cleaved by Dicer proteins, which remove the loop from the pre‐miRNAs structure, generating the miRNA duplex. Finally, this duplex is unwound, and the guide filament of the miRNA is assembled with the RNA‐induced silencing complex (RISC) and is ready to carry out its function by binding the 3’‐untranslated region (3’‐UTR) of mRNA target, repressing the translation or the deadenylation, in the case of partial complementarity with the mRNA, or degradating the mRNA when complementarity occurs, leading to the silencing of the protein synthesis.^5^

Each miRNA can control different genes, and each gene can be regulated by several miRNAs, generating a complex network. More than one thousand miRNAs have been described in humans to regulate about 20%–30% of the translation processes. In the healthy adult human heart and in cardiovascular pathological conditions, miRNA expression has been identified by large sequencing approaches.^6^ The role of various miRNAs has also been defined^7^, ^8^ during embryogenesis, in postnatal cardiac development in the adult healthy heart and in cardiac remodelling.^9^

As demonstrated for many tissues, the human heart is characterized by a subpopulation of adult stem cells with the properties to differentiate into cardiomyocytes, to express stem cell markers with clonogenic features, and with the ability to maintain their functionality in vitro and in vivo. These cells are the cardiosphere‐derived cells (CDCs) and cardiospheres (CSs), which represent the progenitor/stem cell‐enriched population.^10^

CDCs have been tested in clinical trials in which autologous CDCs are isolated from patients, and intracoronarially infused after AMI. Two major studies have demonstrated that their use decreased scar size, improving myocardial function and regeneration.^11^, ^12^ Nevertheless, these cellular treatments showed limited advantages. The cost for the manipulation and culture of the explanted cells in safe conditions calls for new approaches in AMI treatments. A characterization of the genes regulated by miRNAs could be useful in clarifying the mechanisms in the human heart, particularly in the CSs/CDCs counterpart, opening the possibility for their use in therapy. Recently, it was reported that specific miRNAs promote cardiomyocyte proliferation in rats.^13^ Three different groups of genes for pluripotent, transitional and mature cardiomyocyte stage were identified from gene analysis of mRNA expression during the differentiation of induced pluripotent stem cells (hiPSCs) from foetal, adult and hypertensive human heart biopsies. Moreover, a miRNA pattern controlling the stem process into the developmental stage at the transcriptional level was identified.^14^

The aim of our work was to characterize miRNA expression in human heart biopsies and in their CS‐ and CDC‐derived cell populations through microarrays and next‐generation sequencing (NGS) approaches. Initially, we performed the miRNA expression analysis using the Agilent microarray platform and found that a set of miRNA clusters was preferentially expressed in CSs and CDCs compared with the biopsies. To have a wider miRNA profile of the three cell populations, we used an NGS approach: while some groups of miRNAs preferentially expressed in CDCs and CSs found with microarrays were also validated with NGS analysis, other miRNAs were specifically identified as more expressed or downregulated in CDCs and CSs than the biopsies. We identified a positive miRNA expression correlation between the microarray and NGS platforms. Moreover, we identified miRNAs preferentially expressed in CSs and in CDCs, while others were downregulated. Among them, we validated some by real‐time polymerase chain reaction (PCR), and we analysed the correlation between expression and patient age. Moreover, analysis of the NGS results with Diana miRPath software revealed that in CDCs and CSs, miRNAs were involved in the Hippo pathway, the stem cell pathway, the signalling that regulates stem cell pluripotency, and in the signalling involved in arrhythmogenic right ventricular cardiomyopathy.

## 2 MATERIALS AND METHODS

### 2.1 Biopsy specimen processing and cell culture

The isolation, culturing and expansion of cardiac progenitors were carried out on fresh heart biopsies from patients who had undergone extracorporeal circulation. Human surgical auricola biopsies were obtained as part of routine surgical intervention in the Cardiac Surgery and Heart Transplantation Unit at ISMETT, in accordance with institutional guidelines and ISMETT’s Ethics Committee. Informed consent was obtained from all the voluntary participants (IRRB 35/13), as established by the declaration of Helsinki. This study was previously internally approved under protocol number ISMETT.30.09.2013.E.0020458, on 2 October 2013. About 50 biopsies were collected in this study from patients with different diseases: (AVS, aortic valve stenosis; CAD, coronary artery disease; AVRm, aortic valve regurgitation; AAA, ascending aortic aneurysm; BAV, bicuspid aortic valve) (mean age 65 years) (Table 1).

**TABLE 1:** Patients’ summary: table listing the patients enrolled in the study for biological specimen isolation, age, disease and sex are indicated

sample	diseases	Sex	age (years)
cardio 01	AVS+CAD	M	76
cardio 02	AVR+AAA+CAD	M	66
cardio 03	CAD	M	83
cardio 04	CAD	M	57
cardio 05	CAD	M	49
cardio 06	CAD	M	71
cardio 07	CAD	M	55
cardio 08	CAD	F	76
cardio 09	MVR	F	50
cardio 10	AVS (BAV)	M	68
cardio 11	CAD	M	69
cardio 12	CAD	M	67
cardio 13	AAA (BAV)+CAD	M	56
cardio 14	CAD	M	69
cardio 15	AAA (BAV)	F	63
cardio 16	AVR+AAA	M	47
cardio 17	AVS+CAD	F	64
cardio 18	CAD	F	70
cardio 19	AVR	M	64
cardio 20	CAD	F	64
cardio 21	AVS+CAD	M	68
cardio 22	CAD	M	63
cardio 23	AVS	F	82
cardio 24	CAD+AAA	M	78
cardio 25	CAD	M	61
cardio 26	CAD	M	54
cardio 27	AVS	M	73
cardio 28	AVS	F	75
cardio 30	AVS (BAV)	M	74
cardio 31	AVS	F	72
cardio 32	CAD	F	77
cardio 33	AVS (BAV)	M	49
cardio 34	CAD	M	70
cardio 35	CAD	M	64
cardio 36	AVS+CAD	M	76
cardio 37	CAD	M	77
cardio 39	CAD	M	71
cardio 40	AVR+AAA	M	78
cardio 41	AVS	M	75
cardio 42	CAD	M	71
cardio 45	AVR (BAV)+AAA	M	46
cardio 46	AVS	F	71
cardio 47	AVS	F	66
cardio 48	AVS	F	24
cardio 49	AVR (BAV)+AAA	M	50
cardio 50	AVS (BAV)+AAA	M	63

Abbreviations: AAA, ascending aortic aneurysm; AVR, aortic valve regurgitation; AVS, aortic valve stenosis; BAV, bicuspid aortic valve; CAD, coronary artery disease; F, female; M, male.

As previously described,^10^ the biopsies were washed with PBS 1X without Ca++ and Mg++ (Euroclone, Milan, Italy), cleaned from the connective tissue, then mechanically reduced into small fragments and enzymatically digested (0.025%Trypsin‐EDTA, Sigma‐Aldrich, Milan, Italy). The fragments were washed with PBS and seeded on dishes coated with fibronectin (BD Biosciences, Franklin Lakes, NJ) in CDC medium. The CS‐forming cells originated from sprouting from the seeded explant grown over several days. After confluence, the fibroblastic layer cells were harvested by enzymatic digestion (0.05% Trypsin‐EDTA, Sigma‐Aldrich), and the cells were plated on Poly‐D‐Lysine‐coated dishes (Sigma) in CS medium. In these conditions, the CSs grew as floating cell clusters. For expansion, CSs were plated on fibronectin‐treated dishes and expanded as monolayer CDCs, with the following passages (5 times maximum) in CS condition. The CDC medium used was IMDM (Sigma‐Aldrich) plus 20% FCS (Lonza, Basel, Switzerland), Pen/Strep and L‐glutamine (Sigma‐Aldrich). The CS medium was IMDM/DMEM:HAM‐F12 (35%/65%, Sigma‐Aldrich), 4% B27 (Gibco, Milan, Italy), 20 ng/ml bFGF (PeproTech, London, UK), 10 ng/ml EGF (PeproTech), 40 nM Cardiotrophin‐1 (PeproTech), 40 nM L‐thrombin (Sigma‐Aldrich), 3.5% FCS (Lonza), 1% Pen/Strep and 1% L‐glutamine (Sigma‐Aldrich). The cells were also evaluated for specific markers by RT‐PCR.^15^, ^16^

### 2.2 RNA extraction and RT‐PCR

Total RNA was purified by miRNAeasy (Qiagen, Germantown, MD, USA) and reverse‐transcribed using TaqMan UNIVERSAL MMix II (Applied Biosystems, Waltham, MA, USA) for random priming or miRNA‐specific assay reverse transcription. Semi‐quantitative PCR was performed with TaqMan‐validated assays (Applied Biosystems). As endogenous reference gene for cDNA, we chose U6 (#001973) for miRNA. All analyses were carried out in triplicate. Real‐time data were collected using Microsoft Excel and analysed with the following formula: Expression level = 2‐ΔΔCt method.^17^ All experiments were done as independent triplicates and analysed using standard deviation (SD). The p value was obtained with Student's *t* test.

### 2.3 Array analysis

Total RNA was purified by miRNAeasy (Qiagen, Germantown, MD, USA) and retrotranscribed using the Agilent miRNA Labeling Reagent and Hybridization Kit small RNA Agilent—Human miRNA Microarray Kit (V3), and hybridized with the SurePrint Array HD G4470C Microarray Human miRNA Kit (V3) 8x15K containing 866 human and 89 human viral miRNAs (Release 12.0). After hybridization, the microarray was washed according to the manufacturer's protocol. The chips were scanned with an Agilent G2565BA scanner.

Processed logarithmic signal intensities were normalized by quantile normalization method. In each sample, the top‐expressed miRNA covering 90% of the whole miRNA expression was selected, and cluster analysis was performed by considering their union (n = 93). Cluster analysis was implemented with average linkage function. Microarray expression profile data, obtained from the same samples, were compared by computing Pearson's correlation.

### 2.4 NGS analysis

Total RNA was extracted using the miRNeasy Isolation Kit (Qiagen, Hilden, Germany). Sequencing libraries were prepared according to the Illumina Protocol for small RNA (Illumina, San Diego, CA, USA release Feb. 2014), as previously described.^18^ One microgram of total RNA was processed using the small RNA library kit, as described by the manufacturer (Illumina). The library was loaded in an Illumina MiSeq sequencer in a 51 bp single read mode (Illumina). The data obtained from the sequencer were filtered following several criteria. Since the sequence of the adapter is known, a Trimmomatic‐0.33^19^ software was used to trim, from the raw data, the adaptors. The sequence reads were then filtered for quality and clustered to unique sequences to remove redundancy, retaining their individual read count information. Unique sequences 16 nucleotides or more in length were mapped, allowing up to one mismatch on miRNA annotation according to miRBase, using Bowtie 0.12.8^20^ software and HTSeq 0.6.0^21^ software for quantification of the expression of each miRNA. This detects the reads corresponding to known miRNAs, giving an estimation of expression level. Identification of differentially expressed miRNAs was made with the Bioconductor DESeq2^22^ package. Starting from the expression values, the first step was to minimize the effect of the systematic technical variations. Then, a negative binomial distribution model was used to test differential expression in deep sequencing datasets. Only miRNAs with a p value equal to or less than 0.05, and fold change equal to or less than 2.0 and equal to or greater than 2.0 were considered as differentially expressed. Given the critical roles of miRNAs in regulating gene expression and cellular functions, we predicted their putative targets, intersecting results obtained from mirPath v.3.^23^ MirPath provides computationally predicted miRNA gene targets.

### 2.5 Cluster analysis correlation

Up‐/downregulated miRNAs (+/− 2 fold change) were loaded onto DianaTools (mirPath v.3) using the option tarbase v7; we used 6 for the gene intersection analysis on the KEGG pathway. After analysis, we identified the more significant pathways (stem cell, arrhythmogenic and Hippo). The relationship between miRNAs and pathways was represented graphically with Cytoscape, while the relationship between miRNAs and genes was represented with R^24^ (version 3.5.1).

### 2.6 Immunoblotting

Cells and biopsies were lysed with a buffer containing 1% Triton X‐l00, 50 mM HEPES (pH 7.5), 150 mM NaC1, 10% glycerol, 1.5 mM MgCl2, 5 mM EGTA, protease inhibitors (4 mM phenyl methylsulfonyl fluoride and 100 mg/ml aprotinin; Sigma‐Aldrich), phosphatase inhibitors (10 mM sodium orthovanadate and 20 mM sodium pyrophosphate; Sigma‐Aldrich) and processed. For direct immunoblot analysis, we employed 20 μg of total cellular proteins, re‐suspended with 25 μl of loading buffer, boiled for 5 minutes and loaded on SDS‐PAGE for Western blot (WB). The antibodies for WB were used at the condition suggested by the suppliers: mouse anti‐Dvl3 (sc‐271295, 1/500; Santa Cruz Biotechnology, Texas, USA), mouse anti‐Yap1 (sc‐376830, 1/500; Santa Cruz Biotechnology), mouse anti‐Smad1/2/3 (sc‐7960, 1/500; Santa Cruz Biotechnology), rabbit anti‐cyclin D1 (E3P5S #55506, 1/1000; Cell Signaling) and mouse anti‐beta‐actin (sc‐81178, 1/1000; Santa Cruz Biotechnology). The WB has been acquired by the ChemiDoc MP Imaging System (Bio‐Rad Laboratories, Inc., California, USA), and the corresponding bands have been quantified by Image lab 6.1.0 (Bio‐Rad Laboratories, Inc.).

## 3 RESULTS

**FIGURE 1:**
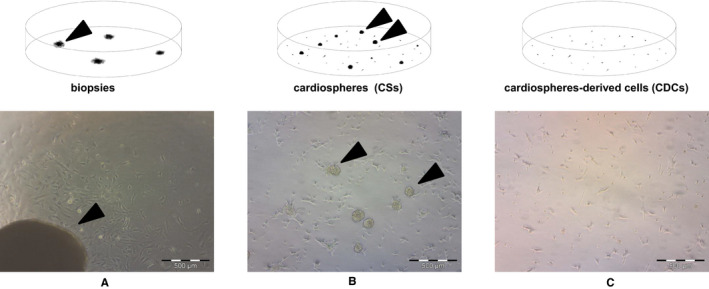
Schematic representation of CS/CDC selection from bioptic specimens. The cardiac tissue fragments were seeded on dishes(A), after cell sprouting these were trypsinized and plated on Poly‐D‐Lysine‐coated dishes to isolate floating CSs (B). CSs floating cell clusters were plated on fibronectin‐treated dishes as CDCs (C)

To evaluate miRNAs involved in cardiac stemness/proliferation phenotypes, we analysed miRNA expression profiles in human heart biopsies and in their derived CSs/CDCs. Cardiac biopsies (Figure 1A) were used to isolate stem/progenitor cells that originated by sprouting in appropriate culture conditions, and a part processed to extract RNA.^15^ The expanded progenitors were used for RNA extraction after 3–6 passages of sequential growth as CSs and CDCs, where CS growth as clusters (Figure 1B), and CDCs showed a fibroblastoid morphology (Figure 1C).

**FIGURE 2:**
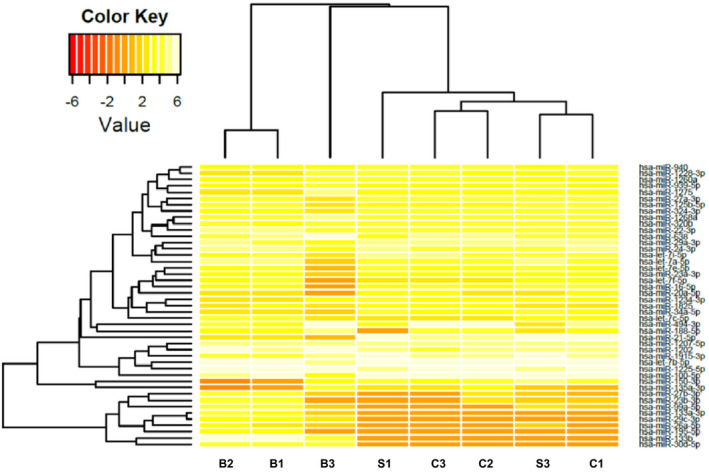
Heat map analysis of the identified miRNAs performed using human CDC “C” Cardiospheres, “S” Spheres and “B” Biopsies with different samples with Agilent microarray (orange: downregulated, white: upregulated).

As a first approach, we evaluated miRNA expression using the Agilent SurePrint Array HD (8x15K) with different samples among biopsies and progenitors (CDCs and CS) (Figure 2). The analysis revealed wide miRNA variation among the diverse samples. Plot analysis displays a clusterization of miRNAs among the different samples: biopsies with biopsies and undifferentiated together, indicating that among the samples there is a conservative pattern depending on the tissue of origin. However, we observed no marked difference among CDCs and CSs, indicating that they are part of the same cell type, with any variation dependant on growth conditions, adherent vs. spheres or growth factors used. In particular, we found various miRNAs strongly downregulated in CSs and CDCs, such as miR‐30, involved in ventricular remodelling,^25^ and in cell proliferation,^26^ or miR‐133, responsible for cardiac differentiative processes^27^ (Figure 2).

To carry out a deeper evaluation of miRNA expression, we performed an NGS analysis with the Illumina platform, and to increase the variability, we used different samples from the microarray analysis.

**FIGURE 3:**
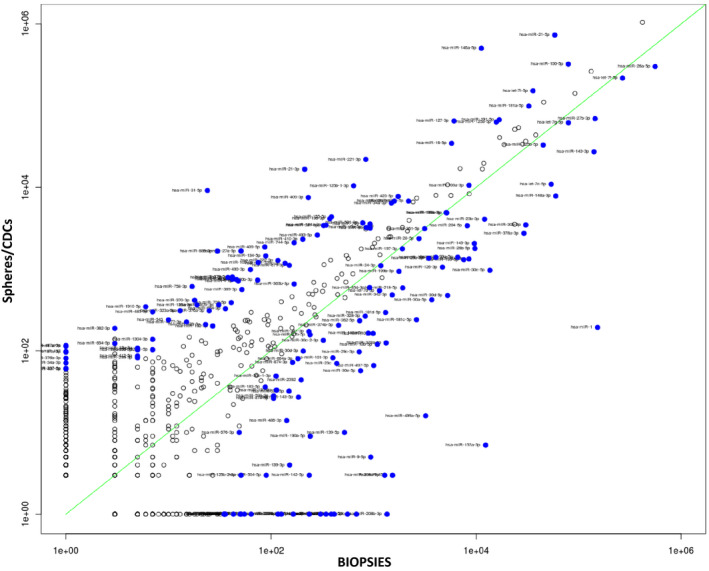
Scatter plot of total miRNA identified by NGS. On the x‐axis, the means of miRNA expression for biopsies are reported, and on the y‐axis, the miRNAs expressed by CDC/CS cells. miRNAs more expressed in progenitors are on the upper left side, and miRNAs more expressed in differentiated heart biopsies are on the lower right. Blue dots represent differentially expressed miRNAs among different samples (fold change, fc=±2; *p*<0.05).

**FIGURE 4:**
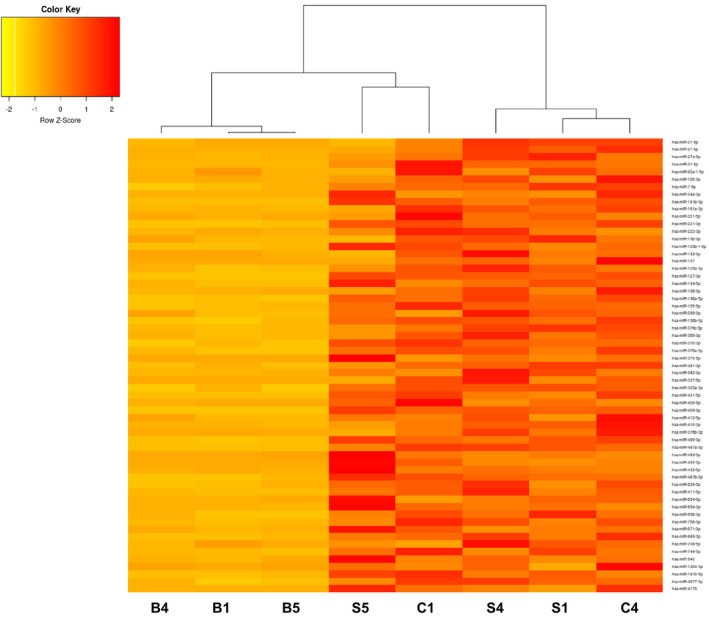
Heat map analysis of miRNAs more abundant in undifferentiated cells, showing the correlation between miRNA expressions obtained from NGS. miRNA expression was hierarchically clustered, showing the association among different cell type: CDC “C” Cardiospheres, “S” Spheres and “B” Biopsies (orange: downregulated, red: upregulated)

**FIGURE 5:**
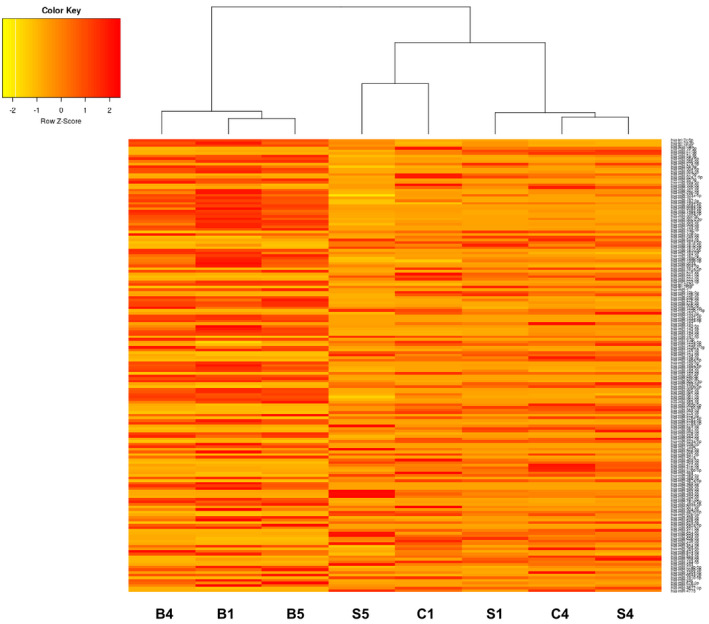
Heat map analysis of miRNAs more abundant in biopsies, showing the correlation between miRNA expressions obtained from NGS. miRNA expression was hierarchically clustered, showing the association among different cell type: CDC “C” Cardiospheres, “S” Spheres and “B” Biopsies (orange: downregulated, red: upregulated)

By NGS, we found various miRNAs that are clearly differentially expressed among the two groups of CDCs/CSs and biopsies (Figure 3). A scatter plot was used to assess miRNA expression variation among sample groups. The values of x‐ and y‐axes in the scatter plot correspond to the average miRNA expression in biopsies and CDC/CS samples, respectively. This analysis clearly illustrates the miRNA expression modifications between undifferentiated and differentiated tissues (Figure 3). NGS analysis results show a correlation with microarray analysis (data not shown). NGS data were further analysed by hierarchical clustering. This approach reveals that the samples cluster based on miRNA expression. We showed through this analysis that progenitors (CDCs and CSs) cluster and that miRNA expression can clearly distinguish undifferentiated cell signature from normal bioptic tissue expression. miRNA expressions observed show a profile with clear correlations among the origin of RNA. Identified miRNA cluster among the different samples define a sharp difference between the differentiated tissue (biopsies) and the proliferating undifferentiated (CDCs/CSs) (Figures 4,5). In particular, progenitor‐overexpressed miRNAs were hierarchically clustered together, while in another analysis miRNAs were downregulated in undifferentiated cells (Figures 4,5). The most upregulated and downregulated miRNAs are summarized in the tables (Table 2: miRNAs upregulated in progenitors; Table 3: miRNAs downregulated in progenitors).

**TABLE 2:** Upregulated in progenitors: selected miRNAs identified by NGS upregulated in progenitors vs. bioptic specimens (p value ≤0.05)

miRNA	Fold change
hsa‐miR−21‐5p	2.7484
hsa‐miR−21‐3p	5.3572
hsa‐miR−27a−5p	4.0918
hsa‐miR−31‐5p	7.4896
hsa‐miR−92a−1‐5p	3.7099
hsa‐miR−100‐3p	3.2196
hsa‐miR−7‐5p	2.9278
hsa‐miR−34a−3p	4.0742
hsa‐miR−181b−3p	4.4813
hsa‐miR−181a−3p	2.4548
hsa‐miR−221‐5p	3.2359
hsa‐miR−221‐3p	3.8665
hsa‐miR−222‐3p	2.6332
hsa‐miR−15b−3p	3.769
hsa‐miR−125b−1‐3p	3.2342
hsa‐miR−132‐3p	2.2501
hsa‐miR−137	4.6336
hsa‐miR−125a−3p	3.3949
hsa‐miR−127‐3p	2.5671
hsa‐miR−134‐5p	3.1897
hsa‐miR−136‐3p	2.5875
hsa‐miR−146a−5p	4.6507
hsa‐miR−155‐5p	2.6301
hsa‐miR−299‐3p	3.3018
hsa‐miR−130b−3p	2.3135
hsa‐miR−376c−3p	2.216
hsa‐miR−369‐3p	2.5734
hsa‐miR−370‐3p	3.903
hsa‐miR−376a−3p	2.8782
hsa‐miR−379‐5p	3.5517
hsa‐miR−381‐3p	2.5755
hsa‐miR−382‐3p	4.5645
hsa‐miR−337‐5p	3.9705
hsa‐miR−323a−3p	4.4264
hsa‐miR−431‐5p	3.8686
hsa‐miR−409‐5p	3.5866
hsa‐miR−409‐3p	4.191
hsa‐miR−412‐5p	3.4294
hsa‐miR−410‐3p	2.6592
hsa‐miR−376b−3p	4.3539
hsa‐miR−485‐3p	4.4034
hsa‐miR−487a−3p	4.8424
hsa‐miR−493‐5p	2.4696
hsa‐miR−493‐3p	3.2197
hsa‐miR−432‐5p	3.6742
hsa‐miR−487b−3p	2.5732
hsa‐miR−629‐5p	2.202
hsa‐miR−411‐5p	3.1905
hsa‐miR−654‐5p	3.8602
hsa‐miR−654‐3p	2.3029
hsa‐miR−656‐3p	3.6479
hsa‐miR−758‐3p	4.2105
hsa‐miR−671‐3p	2.015
hsa‐miR−889‐3p	4.7122
hsa‐miR−708‐5p	2.5322
hsa‐miR−744‐5p	2.8278
hsa‐miR−543	3.5297
hsa‐miR−1304‐3p	3.3442
hsa‐miR−1910‐5p	4.7108
hsa‐miR−4677‐3p	3.0669
hsa‐miR−4775	4.7372

**TABLE 3:** Downregulated in progenitors: selected miRNAs identified by NGS downregulated in progenitors vs. bioptic specimens (p value ≤0.05)

miRNA	Fold change
hsa‐let‐7c‐5p	−3.0333
hsa‐miR‐26b‐5p	−3.2806
hsa‐miR‐30a‐5p	−3.9109
hsa‐miR‐30a‐3p	−2.3937
hsa‐miR‐95‐3p	−4.2943
hsa‐miR‐99a‐5p	−3.1569
hsa‐miR‐101‐3p	−2.9907
hsa‐miR‐29b‐3p	−2.1912
hsa‐miR‐107	−3.469
hsa‐miR‐208a‐5p	−7.5547
hsa‐miR‐208a‐3p	−8.5667
hsa‐miR‐148a‐5p	−3.2549
hsa‐miR‐148a‐3p	−3.8122
hsa‐miR‐30c‐5p	−4.6598
hsa‐miR‐30c‐2‐3p	−2.0636
hsa‐miR‐30d‐5p	−4.2635
hsa‐miR‐139‐5p	−6.0925
hsa‐miR‐139‐3p	−5.5364
hsa‐miR‐10b‐5p	−4.1262
hsa‐miR‐181c‐5p	−4.2359
hsa‐miR‐183‐5p	−2.4598
hsa‐miR‐187‐3p	−4.4679
hsa‐miR‐203a	−5.6513
hsa‐miR‐204‐5p	−2.0446
hsa‐miR‐218‐5p	−2.6125
hsa‐miR‐223‐3p	−5.8025
hsa‐miR‐1	−10.707
hsa‐miR‐23b‐3p	−2.4521
hsa‐miR‐30b‐5p	−4.0408
hsa‐miR‐133a‐5p	−6.7325
hsa‐miR‐133a‐3p	−11.9
hsa‐miR‐135a‐5p	−7.3079
hsa‐miR‐142‐5p	−6.1027
hsa‐miR‐143‐5p	−3.516
hsa‐miR‐143‐3p	−3.2519
hsa‐miR‐145‐5p	−3.1335
hsa‐miR‐9‐5p	−8.1911
hsa‐miR‐125b‐2‐3p	−4.0425
hsa‐miR‐126‐3p	−3.0177
hsa‐miR‐150‐5p	−6.2097
hsa‐miR‐190a‐5p	−5.6668
hsa‐miR‐195‐5p	−3.3999
hsa‐miR‐195‐3p	−2.6979
hsa‐miR‐29c‐5p	−8.3012
hsa‐miR‐29c‐3p	−3.761
hsa‐miR‐30c‐1‐3p	−2.2355
hsa‐miR‐30e‐5p	−4.4932
hsa‐miR‐30e‐3p	−3.5164
hsa‐miR‐362‐5p	−2.4459
hsa‐miR‐363‐3p	−4.0285
hsa‐miR‐372‐3p	−4.8576
hsa‐miR‐378a‐5p	−4.4058
hsa‐miR‐378a‐3p	−4.2366
hsa‐miR‐328‐3p	−2.6698
hsa‐miR‐342‐3p	−2.5186
hsa‐miR‐338‐3p	−3.9172
hsa‐miR‐133b	−7.1304
hsa‐miR‐20b‐5p	−3.9172
hsa‐miR‐451a	−8.8595
hsa‐miR‐486‐3p	−5.3778
hsa‐miR‐489‐3p	−4.4628
hsa‐miR‐490‐5p	−7.9944
hsa‐miR‐490‐3p	−7.4075
hsa‐miR‐497‐5p	−4.6885
hsa‐miR‐181d‐5p	−3.0345
hsa‐miR‐499a‐5p	−8.9654
hsa‐miR‐504‐5p	−4.6711
hsa‐miR‐598‐3p	−2.7942
hsa‐miR‐628‐5p	−3.3797
hsa‐miR‐642a‐5p	−4.0123
hsa‐miR‐651‐5p	−4.5977
hsa‐miR‐874‐5p	−2.6084
hsa‐miR‐208b‐3p	−9.282
hsa‐miR‐664a‐3p	−2.1935
hsa‐miR‐23c	−3.2984
hsa‐miR‐676‐3p	−3.3675
hsa‐miR‐2392	−3.2562

**FIGURE 6:**
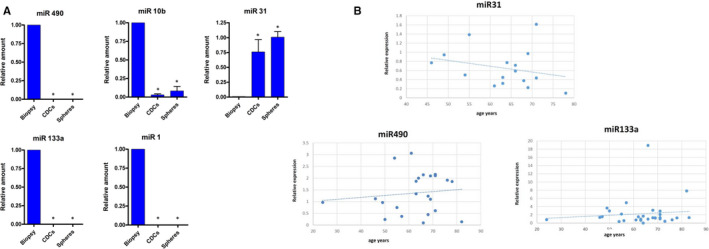
RT‐PCR analysis on selected miRNAs. (A) miR‐1, miR‐133a, miR‐10b, miR‐490 and miR‐31 expression were evaluated in human heart CDCs, CSs and biopsies (all reported experiments were performed in triplicate on three different samples *p value ≤0.05). (B) Correlation between patient age and miR‐490 (ρ=0.119543, p value=0.000188258), miR‐133a (ρ=0.112118341 p value=0.00000001) and miR‐31 (ρ=−0.2608, p value=0.005157) expressions.

We evaluated the reliability of our NGS results by choosing some miRNAs to check the differential expression through RT‐PCR analysis (Figure 6A). In particular, we observed that miR‐1, miR‐10b, miR‐133a and miR‐490 were downregulated in CDCs and CSs, while they were highly expressed in differentiated human heart tissues. The expression of miR‐31 was higher in CDC and CS samples (Figure 6A). Notably, the expression of the miR‐133 family, as assessed in microarray analysis, was remarkable in its role in human heart development.^28^ We also tested whether the differential expression was correlated with patient age. We evaluated miR‐490, miR‐31 and miR‐133a expression in different biopsies. Our analysis revealed that miR‐31, which found more expressed in undifferentiated progenitors, has an inverse correlation with patient age, while the miRNAs overexpressed in differentiated tissues, such as miR‐133a and miR‐490, increase with the patient's age (Figure 6B).

**FIGURE 7:**
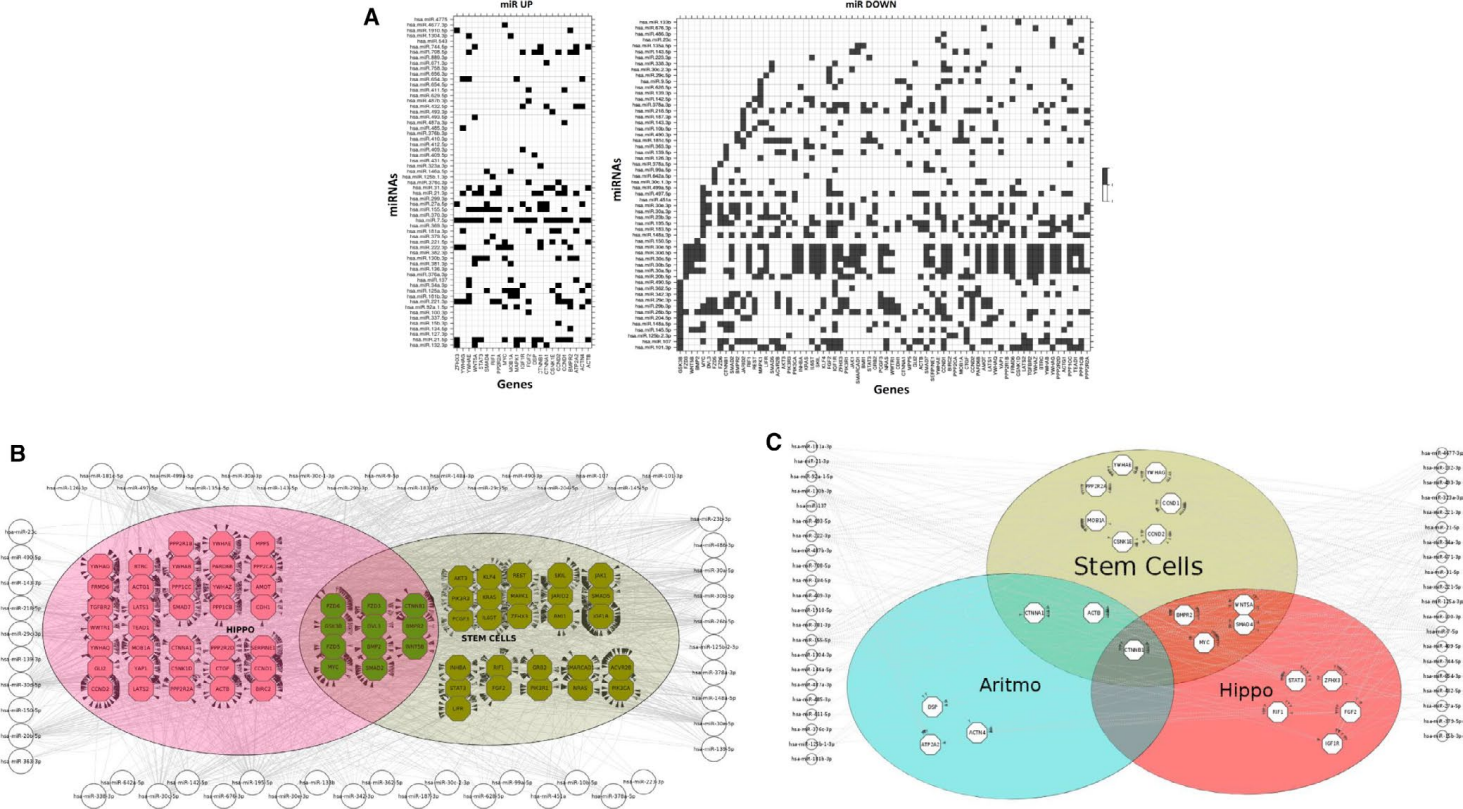
miRNA target analysis. (A) Heat map showing differentially expressed miRNAs versus target gene predicted. Using DIANA MirPath, we provided a graphical overview of selected progenitor's upregulated miRNAs (left) and progenitor's downregulated miRNAs (right). Pathway clustering analysis was also performed for the biological pathways using KEGG pathways as in the heat map. miRNAs in downregulated progenitors (B) and upregulated (C) were considered.

To better understand the implications of miRNA expression in the repair processes and in stemness maintenance, we analysed differentially expressed miRNAs with Diana mirPath to find a possible correlation between miRNA and target genes associated with their regulation. In particular, we selected the miRNAs more than twofold upregulated and downregulated in stem cells/progenitors, with a p value <0.05 among all analysed samples. The miRNAs were used to perform a gene intersection analysis through Tarbase (v7). Many of the genes regulated by selected miRNAs are involved in proliferation supporting the reliability of our results (Figure 7A). Furthermore, miRNA gene targeting analysis software showed specific pathways on the KEGG database. Downregulated miRNAs appear to modulate many genes belonging to the Hippo pathway and the signalling pathways regulating pluripotency of stem cells (Figure 7B). The same pathways have been found by the analysis of upregulated miRNAs that also target genes involved in the arrhythmogenic right ventricular cardiomiopathy (ARVC) pathway (Figure 7C). The regulation of such specific pathways offers the idea of a precise signature in post‐transcriptional regulation that leads to a strict control of cardiac progenitor proliferation and differentiation.

**FIGURE 8:**
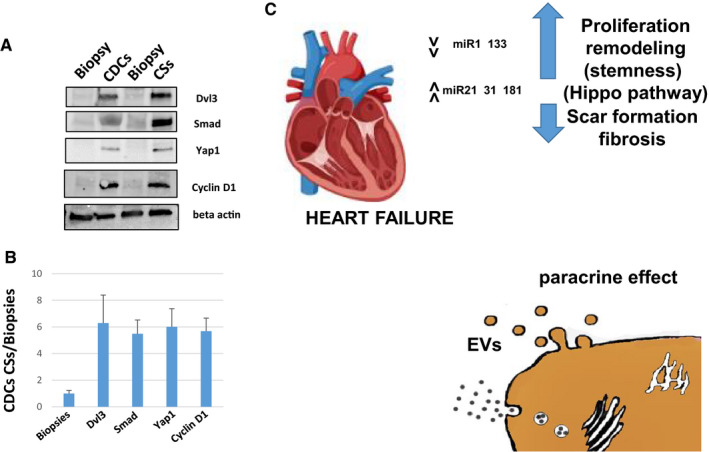
Western blot analysis on Hippo pathway proteins. (A) Cell extracts from Biopsies, CDCs and CSs were used to evaluate Dvl3, SMAD 1/2/3, Yap1 and cyclin D1, the beta‐actin as control. All proteins appear clearly overexpressed in CDCs/CSs and not in the corresponding biopsies (two out of four sample patients have been shown). (B) Intensity ratio of the indicated proteins: CDCs and CSs vs Biopsies (the analysis has been carried on four different samples in triplicate p value ≤0.05) (C) Schematic representation of miRNA’s mechanism of action as illustrated in the Discussion

In order to evaluate the relationship among the differentially expressed miRNAs and the specific downstream‐targeted pathway, we analysed by WB some of the key genes belonging to the Hippo pathway. In particular, we found Yap1 clearly differentially expressed in proliferating progenitors vs. biopsies (Figure 8). As expected, other Hippo pathway members were found to be differentially expressed, such as Smad proteins (Figure 8). Likewise, dishevelled 3 (Dvl3), a scaffolding protein implicated also in Wnt and pathway regulation,^29^ is clearly upregulated in CDCs and CSs (Figure 8). Hippo downstream signalling, such as cyclin D1,^30^ which promotes proliferation appear upregulated in undifferentiated progenitors (Figure 8).

## 4 DISCUSSION

The ability of the myocardium to regenerate after AMI was demonstrated more than twenty years ago with the observation in mice models in which delivered bone marrow (BM) progenitors can ameliorate the outcome by generating myocardial cells.^31^ Furthermore, mobilized BM in MI patients showed an improvement in function and survival.^32^ The use of human cardiac progenitors in the CADUCEUS^11^ and PERSEUS^12^ trials showed limited benefit in using cardiac progenitors (CDCs) for MI treatment. These studies found scar mass reduction, with increased heart mass and contractive activity. However, the following clinical trials were not satisfying,^33^ showing a low expansion efficiency of transplanted cells due to loss of cell anchorage and a short life of the transplanted cells.^34^ To circumvent these limitations, scaffolds embedded with cells, or even the administration of vesicles containing proteins, DNA molecules and miRNAs released by cardiovascular progenitor cells were also applied, showing that other factors could contribute to the regeneration of the infarcted heart.^35^ In this scenario, miRNAs may represent a promising therapeutic tool because they are easily synthesized and can be proficiently encapsulated in particles/exosomes because of their small size. For this reason, we investigated possible mechanisms from molecular signature results that could determine the proliferative ability of cardiac progenitors. In our study, through microarray and NGS analysis, we aimed to clarify the miRNA expression profile in the CDCs and CSs compared with the differentiated tissue from the same patient.

We found a different miRNA expression pattern in our CDCs/CSs compared with the biopsies, identifying several miRNAs that can be responsible for the maintenance of an undifferentiated state. Notably, miR‐1 and miR‐133 were strongly downregulated in our CDC/CS cells. From previous studies, both miRNAs have been found to play a role in the differentiation process, as well as to have a specific function in remodelling events^36^; moreover, they were upregulated during cardiomyocyte differentiation.^14^ It has been reported that miR‐133a‐deficient hearts show increased and aberrant cardiomyocyte proliferation throughout the atria and ventricles; this latter result potentially explains the development of a lethal ventricular septal defect in miR‐133 knockout mice, where miR‐133a regulates several transcription factors involved in cell cycle control such as cyclin D1, which we found overexpressed in progenitor cells.^28^ This cyclin is also regulated by miR‐1,^37^ and its overexpression promotes cardiomyocyte commitment in human cardiovascular progenitors through the suppression of WNT and FGF signalling pathways.^38^ The involvement of miRNAs in the FGF pathway opens an important question on the balance between the proliferative activity in cardiac repair after MI and the fibrotic processes. The FGF pathway has been described as influenced by miR‐133, which suppresses atrial remodelling.^39^ As here observed, miR‐133 is downregulated in undifferentiated proliferation progenitors, and its expression increases with patient age, most likely accounting for the reduced capacity to repair in aged hearts.^40^, ^41^ FGF signalling in the heart acts in a paracrine mode, influencing development, in a healthy or pathologic status. We found that undifferentiated proliferating progenitors show miR‐31 increased expression. miR‐31 has been shown to influence cell migration and proliferation by modulating different genes^42^; however, in mice models, miR‐31 overexpression promotes adverse cardiac remodelling and dysfunction in ischaemic heart disease.^43^ Cardiac remodelling is a major cause of morbidity and mortality in heart failure, where fibrosis replacement is a critical issue. For this reason, a better comprehension of the fine regulation to maintain a proliferative activity in cardiac resident cells, without an increase in fibrotic transition, is needed. It is remarkable to consider that the miRNAs also act in a paracrine way. It has been demonstrated that they can play a role when secreted in exosomes in various processes (eg in angiogenesis^44^). The miR‐21 (Table 2), which we found upregulated in progenitors, has been shown to have a protective effect in rats against myocardial infarction.^45^ Moreover, cardiomyocyte‐derived conditioned medium can exert an action, miR‐21‐dependent, in reducing infarct size in MI induced rats.^45^ Likewise, miR‐181 has been found to rescue deterioration of cardiac function in post‐MI models by suppressing the Aldo–MR pathway.^46^ This miRNA has been found to be secreted in exosomes in MSCs models.^47^ In a different model, it has been demonstrated that miR‐31 is secreted in plasma exosomes^48^; therefore, further studies could evaluate the miR‐31 or other miRNAs effects on cardiac remodelling when loaded on exosomes.

Our results underscore a regulation of Hippo pathway genes by various miRNAs identified and differentially expressed among progenitors (CDCs/CSs) vs. bioptic tissues. In particular, we found a specific involvement of the Hippo pathway in progenitors that can be responsible for a higher expression of specific proteins such as Yap1, Smad, Dvl3 and cyclin D1. It has been demonstrated that Yap1 is necessary for regeneration in neonatal injured heart mice.^49^, ^50^ Its interaction with β‐catenin remarks the connection among Hippo and Wnt signalling in the regulation of cardiomyocyte proliferation and heart size controls.^51^ Another association between the two pathways is represented by the interaction Yap/Dvl.^29^ Dvl3, which we found upregulated in progenitors, is responsible for heart formation in mice models.^52^ Furthermore, Robinow syndrome has been linked to a mutation on the Dvl3 gene^53^ (3q27.1 omim.org/entry/601368) with various heart developmental defects (ventricular septal defect, patent foramen ovale, pulmonary atresia, hypoplastic right heart, and tricuspid regurgitation) (omim.org/entry/616894, revised by Gentzel et al.^54^). Our data on regulation of the Hippo pathway reveal a connection among progenitor proliferation miRNA signature and the in vivo regulation of the heart development. Further analysis could open new avenues to find innovative approaches and suggest functional investigations in order to identify mechanisms responsible for the myocardial repair during the proliferative activity of undifferentiated cells. In particular, narrowed studies based on our miRNA expression can be conducted in post‐MI patients to identify the most promising for therapeutic or diagnostic purposes.

## CONFLICT OF INTEREST

The authors declare that they have no conflict of interest.

## AUTHOR CONTRIBUTION

**gioacchin iannolo:** Conceptualization (lead); Data curation (lead); Formal analysis (lead); Investigation (lead); Project administration (lead); Supervision (lead); Writing‐original draft (lead); Writing‐review & editing (lead). **maria rita sciuto:** Data curation (equal); Writing‐original draft (equal); Writing‐review & editing (equal). **Nicola Cuscino:** Data curation (supporting); Formal analysis (supporting); Software (supporting). **Claudia Carcione:** Formal analysis (supporting); Validation (supporting). **claudia coronnello:** Data curation (supporting); Formal analysis (supporting); Software (supporting). **cinzia maria chinnici:** Investigation (supporting). **giuaseppe maria raffa:** Resources (supporting). **michele pilato:** Investigation (supporting). **Pier Giulio Conaldi:** Funding acquisition (lead); Investigation (supporting).

## Data Availability

The data that support the findings of this study are available from the corresponding author upon reasonable request.
